# Differential gene expression and Hog1 interaction with osmoresponsive genes in the extremely halotolerant black yeast *Hortaea werneckii*

**DOI:** 10.1186/1471-2164-8-280

**Published:** 2007-08-16

**Authors:** Tomaž Vaupotič, Ana Plemenitaš

**Affiliations:** 1 Institute of Biochemistry, University of Ljubljana Faculty of Medicine, Vrazov Trg 2, SI-1000 Ljubljana, Slovenia

## Abstract

**Background:**

Fluctuations in external salinity force eukaryotic cells to respond by changes in the gene expression of proteins acting in protective biochemical processes, thus counteracting the changing osmotic pressure. The high-osmolarity glycerol (HOG) signaling pathway is essential for the efficient up-regulation of the osmoresponsive genes. In this study, the differential gene expression of the extremely halotolerant black yeast *Hortaea werneckii *was explored. Furthermore, the interaction of mitogen-activated protein kinase HwHog1 and RNA polymerase II with the chromatin in cells adapted to an extremely hypersaline environment was analyzed.

**Results:**

A cDNA subtraction library was constructed for *H. werneckii*, adapted to moderate salinity or an extremely hypersaline environment of 4.5 M NaCl. An uncommon osmoresponsive set of 95 differentially expressed genes was identified. The majority of these had not previously been connected with the adaptation of salt-sensitive *S. cerevisiae *to hypersaline conditions. The transcriptional response in hypersaline-adapted and hypersaline-stressed cells showed that only a subset of the identified genes responded to acute salt-stress, whereas all were differentially expressed in adapted cells. Interaction with HwHog1 was shown for 36 of the 95 differentially expressed genes. The majority of the identified osmoresponsive and HwHog1-dependent genes in *H. werneckii *have not been previously reported as Hog1-dependent genes in the salt-sensitive *S. cerevisiae*. The study further demonstrated the co-occupancy of HwHog1 and RNA polymerase II on the chromatin of 17 up-regulated and 2 down-regulated genes in 4.5 M NaCl-adapted *H. werneckii *cells.

**Conclusion:**

Extremely halotolerant *H. werneckii *represents a suitable and highly relevant organism to study cellular responses to environmental salinity. In comparison with the salt-sensitive *S. cerevisiae*, this yeast shows a different set of genes being expressed at high salt concentrations and interacting with HwHog1 MAP kinase, suggesting atypical processes deserving of further study.

## Background

When a living organism is subjected to extreme environmental conditions for an extended period of time, an adaptive response may become crucial for its continued existence. The response of eukaryotic cells such as yeast to environmental stress involves complex changes in gene expression which subsequently lead to various metabolic responses to induce adaptation to the new conditions. Fluctuating external osmolarity, like changes in salt concentration, leads to altered transcription of many responsive genes in an effort to counteract the stress with the activity of their protein products. One of the earliest protective biochemical responses is the biosynthesis and accumulation of glycerol as an osmolyte via the activation of corresponding genes. The resulting glycerol accumulates in the cytosol and leads to increased internal osmolarity, thus restoring the osmotic gradient between the cells and their environment [1]. In the salt-sensitive yeast *Saccharomyces cerevisiae*, the hyperosmotic stress caused by 0.4 M NaCl leads to the transient transcriptional induction of more than 1500 genes, as a consequence of simultaneous action of the general stress response pathway together with the high-osmolarity glycerol (HOG) mitogen-activated protein kinase (MAPK) signaling pathway [2]. A functional HOG pathway is essential for the efficient up-regulation of the vast majority of genes in response to hyperosmotic conditions [3,4]. The terminal MAPK, Hog1, accumulates in the nucleus within minutes of exposure to high salt concentrations [5], whereupon it phosphorilates and activates the HOG-specific transcription factors Sko1 [6] and Smp1 [7], or recruits Hot1 [8] and the general stress-response transcriptional activators Msn1, Msn2 and Msn4 [9,10] to the promoters of osmoinducible genes. Findings that Hog1 could be an integral part of the upstream activation complex, targeting not only the activators but also components of the general transcription machinery, such as RNA polymerase II [11,12] together with Hog1-guided recruitment of Rpd3 histone deacetylase to the chromatin [13], have highlighted the additional level of complexity in the regulation of gene expression during hyperosmotic conditions.

To date, studies on hyperosmotic adaptation and salt tolerance in fungal species have been largely performed with the salt-sensitive model organism *S. cerevisiae*, for reasons of experimental convenience. However, the cellular machinery of *S. cerevisiae *is not adapted to the extreme hyperosmolar pressure caused by a salty environment with more than 1–2 M NaCl concentration. Therefore the specially adapted extremely halotolerant yeast-like fungus *Hortaea werneckii *represents a novel eukaryotic organism for studying cellular responses to extremely elevated environmental salinity. This naturally osmoadaptable species was first isolated from hypersaline crystallizer ponds in salterns [14], where NaCl concentration fluctuates from 0.5 M up to saturate solution levels (6 M). The increase in surrounding salt concentration is accompanied by increased intracellular glycerol accumulation in *H. werneckii *[15]. The glycerol accumulation suggested the activation of the HOG-like pathway. The MAPK HwHog1 was later identified, showing its highest activity at a concentration of 4.5 M NaCl [16]. Many cellular and physiological differences were observed between *H. werneckii *cells growing in the extremely hypersaline environment of 4.5 M NaCl and those growing in a moderate salinity of 3 M NaCl, which has been assigned as the optimal metabolic condition for *H. werneckii *[15,17-19]. Therefore the characterization of the differential transcriptional response and identification of HwHog1-target genes in *H. werneckii *when under extreme hypersaline conditions could provide new insight into eukaryotic saline-response genetics.

In this study we have identified a set of differentially expressed genes in hyperosmotically adapted *H. werneckii*. Expression profiles of the genes were determined for both hyperosmotically-adapted and hyperosmotically-stressed *H. werneckii *cells. The identified genes were tested for interaction with HwHog1 and RNA polymerase II. Here we report a new insight into the differential expression of osmoresponsive genes in an extremely halotolerant eukaryotic microorganism and suggest a role for these genes in adaptive metabolism.

## Results and Discussion

Since *H. werneckii *occupies a different ecological niche than *S. cerevisiae*, it is not surprising that these yeasts perceive hyperosmotic environments differently. In contrast to *S. cerevisiae*, *H. werneckii *can actively grow in a wide range of external salt concentrations, which shows that the cells are also well adapted to an extremely hypersaline environment. Differentially expressed genes in *H. werneckii *cells grown in different salinities therefore represent the transcriptional response of the adapted cells, rather than the stress response. Previous work has shown differences in the activation of HwHog1 between the halotolerant *H. werneckii *and *S. cerevisiae *[16]. In the present work, we tried to address two questions: (i) what is the transcriptional response of *H. werneckii *adapted to a hypersaline environment compared with transcription under optimal growth conditions, and (ii) whether and how the major HOG pathway effector MAPK HwHog1 is associated with the expression of osmoresponsive genes in *H. werneckii*.

### Bioinformatical characterization of the Hw4.5-3 subtracted library revealed over-representation of *H. werneckii *osmoresponsive genes in functional groups of metabolism and energy

It is a special challenge to identify a relatively large group of differentially-expressed genes that are specifically connected with the response to a certain environmental stimulus in an organism, without the availability of a sequenced genome. A suppression subtractive hybridization (SSH) cDNA library was prepared to study the differential expression of genes related to halotolerance in *H. werneckii *between cells growing in optimal salinity (3 M NaCl) and cells growing in extreme salinity (4.5 M NaCl). A mirror orientation selection (MOS) method [20] and differential cross-screening of the SSH-clones were employed after SSH to augment the subtracted library for differentially expressed genes. A total of 1088 colonies were subjected to highly stringent differential screening. According to this selection, 170 clones (15.6%) from the Hw4.5-3 subtracted library were strong candidates for differentially expressed genes. The nucleotide sequences were analyzed and their putative functions identified by searching BLASTX. As a result, a total of 95 (55.8%) unique singletons and assembled sequences were identified (see Table 1). Redundancy-check analysis (Table 2) revealed that the 10 most abundant ESTs occupied 35.8% of the Hw4.5-3 subtraction library. A comparison with the GenBank database revealed that 74 of the 95 cDNAs (77.8%) had a high degree of sequence similarity (*E*-value ≤ 2.00E-11) with genes from other organisms of fungal origin. From the remaining 24 sequences (25.3%), 11 sequences (11.6%) could be identified with a low degree of similarity and 13 sequences (13.7%) with weak or no similarity (*E*-value cut off at 1.00E-02) to genes from other organisms. The names for the identified sequences were assigned according to the *Saccharomyces *Genome Database [21] standard names of the closest orthologs from *S. cerevisiae *with the prefix *Hw *for *Hortaea werneckii *(Table 1). For unidentified genes, weakly similar genes or genes without known orthologs in *S. cerevisiae*, the names were assigned as *SOL *followed by a consecutive number in our arbitrary library of *H. werneckii *hyperosmolarity-induced expressed sequence tags (ESTs) [15]. The putative functions of the ESTs with *E*-values lower than 10^-5 ^(Table 1) were categorized as suggested by the MIPS database FunCat functional annotation scheme based on *S. cerevisiae *classification [22,23]. For eight ESTs without a corresponding *S. cerevisiae *ortholog, a matched ortholog was found in other organisms and they were functionally classified by literature (Table 1, numbers in brackets). As can be seen in Table 3, general metabolism proteins (31.6% of total unique transcripts), followed by energy production (23.3%), were the most common categories among the 16 functional categories. Of 13 unclassified *SOL *genes, *SOL10*, *SOL19*, *SOL20*, *SOL21*, *SOL23*, *SOL24*, *SOL26 *and *SOL29 *showed very weak similarity with other *S. cerevisiae *genes as aligned by a Wu-BLAST2 search, whereas *SOL18*, *SOL 22*, *SOL25*, *SOL27 *and *SOL28 *were completely unrelated to known genes from databases. These transcripts might thus be specifically related to the adaptation capability of *H. werneckii *to extremely high salt concentrations. The most unexpected discovery among differentially expressed ESTs from the Hw4.5-3 subtracted library was the identification of the *SOL13 *gene, which shows the highest similarity with opsins from two other *Dothideomycetes *fungi, *Phaeosphaeria nodorum *and *Leptosphaeria maculans*, respectively. *L. maculans *rhodopsin represents the first proven case of a light-driven transmembraneproton translocation by retinal-binding protein from a fungal organism [24], suggesting that some fungal classes may use the rhodopsin-based bioenergetic pathway previously observed only in prokaryotes.

**Table 1:** Differentially expressed genes in *H. werneckii *adaptation to 3 M NaCl or 4.5 M NaCl. BLASTX matches of EST clones derived from the Hw4.5-3 subtraction library and their functional categorizations based on MIPS are collected. Similarities with probability <10^-5 ^were regarded as being significant, and others as not determined (*ND*). Genes selected for the expression and chromatin immunoprecipitation analyses are printed in bold. ^a^GenBank accession number. ^b^Numbers indicate the functional groups (see Table 3). Numbers in brackets correspond to literature-based arbitrarily-assigned functional categories for ESTs with orthologs in organisms other than *S. cerevisiae*. ^c^Fold induction (fI, positive value) or fold repression (fR, negative value) in 4.5 M NaCl- *vs*. 3 M NaCl-adapted *H. werneckii *cells. Numbers indicated are mean values of three independent RT-PCR experiments, and the representative gels are shown in figures 1 and 2.

GenBank.^a^	Gene	Characteristic or description	Functional category^b^	fI/f R^c^
DQ822684	***HwAGP1***	Amino acid permease	20.	**3.3**
DQ822624	***HwATP1***	Mitochondrial ATPase alpha-subunit	2. 20. 34.	**1.8**
DQ822625	***HwATP2***	Mitochondrial ATPase beta-subunit	2. 20. 34. 40.	**4.0**
DQ822626	***HwATP3***	Mitochondrial ATPase gamma-subunit	2. 20. 34.	**2.3**
DQ822606	***HwBMH1***	14-3-3 protein	1. 10. 16. 30. 40. 43.	**1.8**
DQ822642	***HwCDC3***	Septin complex component	10. 16. 34. 40. 42. 43.	**-3.3**
DQ822620	***HwCIT1***	Mitochondrial citrate synthase	1. 2. 42.	**4.6**
DQ822622	***HwCOB1***	Cytochrome b	2.	**5.2**
DQ822621	***HwCOX1***	Cytochrome c oxidase subunit I	2. 20.	**2.5**
DQ822687	***HwCYT1***	Cytochrome c1	2. 20.	**3.6**
DQ822643	***HwDBP2***	RNA helicase	1. 11.	**-4.2**
DQ822641	***HwECM33***	Extracellular matrix 33 protein	10. 42. 43.	**2.0**
DQ822657	***HwEFT2***	Eukaryotic translation elongation factor 2 (eEF-2)	12.	**1.9**
DQ822685	***HwELF1***	Transcription elongation factor	10. 11. 40	**7.6**
DQ822613	***HwERV25***	p24 component of the COPII-coated vesicles	20.	**9.4**
DQ822619	***HwFAS1***	Mitochondrial acyl-carrier protein	1.	**3.1**
DQ822689	***HwFRE7***	Ferric-chelate reductase-7 transmembrane component	20. 34.	**10.5**
DQ822656	***HwFUN12***	Eukaryotic translation initiation factor 5B (eIF-5B)	12.	**3.4**
DQ822655	*HwGAL10*	UDP-glucose 4-epimerase	1.	*ND*
DQ822610	***HwGDH1***	NADP(+)-specific glutamate dehydrogenase	1. 2. 16.	**7.5**
DQ822654	***HwGND2***	6-phosphogluconate dehydrogenase	1. 2. 16.	**4.1**
DQ822631	***HwGUT2***	FAD-dependent glycerol-3-phosphate dehydrogenase	1. 2. 42.	**3.0**
DQ822645	*HwHHF1*	Histone H4	10. 11. 16.	*ND*
DQ822614	***HwHSP82***	Heat shock protein 90	1. 2. 10. 14. 32. 34. 43.	**5.1**
DQ822646	***HwHTA1***	Histone H2A	10. 11. 16.	**-2.6**
DQ858164	***HwIRE1***	Serine-threonine protein kinase and endoribonuclease	1. 11. 14. 30. 32.	**2.1**
DQ822615	***HwKAR2***	Endoplasmic reticulum luminal chaperone	1. 10. 14. 16. 20. 32. 41. 42.	**2.3**
DQ822627	***HwKGD2***	Dihydrolipoamide succinyltransferase	1. 2.	**1.8**
DQ822629	***HwLSC2***	Mitochondrial succinyl-CoA ligase beta-chain	1. 2. 16.	**2.7**
DQ822630	***HwMDH1***	Mitochondrial malate dehydrogenase	1. 2. 42.	**3.4**
DQ858163	***HwMET14***	ATP adenosine-5'-phosphosulfate 3'-phosphotransferase	1.	**3.4**
DQ822607	***HwMET17***	Cystein synthase	1.	**8.2**
DQ822605	***HwMET6***	Cobalamin-independent methionine synthase	1.	**3.2**
DQ822628	***HwMIR1***	Mitochondrial phosphate transport protein	20. 34.	**8.4**
DQ822649	***HwNHP6A***	High mobility group protein A	10. 11. 40. 43.	**4.5**
DQ822623	***HwNUC1***	Mitochondrial nuclease	1. 10.	**3.2**
DQ822635	***HwOPI3***	Unsaturated phospholipid methyltransferase	1.	**9.7**
DQ822612	***HwPDI1***	Protein disulphide isomerase precursor	14. 20.	**2.1**
DQ858158	***HwPGK1***	3-phosphoglycerate kinase	1. 2. 16.	**6.4**
DQ822634	***HwPMA2***	Plasma membrane proton-exporting ATPase	2. 20. 34.	**7.9**
DQ822651	***HwPRY1***	Pathogenesis-related protein precursor	34.	**7.5**
DQ822686	***HwPUF1***	Serine rich pumilio family RNA binding domain protein	1. 10. 11. 16.	**4.6**
DQ822688	***HwRAD16***	SWI/SNF family DEAD/DEAH box helicase	10. 32.	**4.9**
DQ822660	*HwRPL10*	60S ribosomal protein 10	10. 12. 14.	*ND*
DQ822672	*HwRPL16A*	60S ribosomal protein 16A	12. 16.	*ND*
DQ822664	*HwRPL22A*	60S ribosomal protein 22A	12.	*ND*
DQ822670	*HwRPL2B*	60S ribosomal protein 2B	12.	*ND*
DQ822671	*HwRPL3*	60S ribosomal protein 3	12.	*ND*
DQ822669	***HwRPL6A***	60S ribosomal protein 6A	12. 16.	**2.6**
DQ822661	*HwRPL7B*	60S ribosomal protein 7B	12.	*ND*
DQ822617	***HwRPN2***	26S proteasome regulatory particle subunit	11. 40. 30.	**2.2**
DQ822673	*HwRPS10B*	40S ribosomal protein 10B	12.	*ND*
DQ822666	*HwRPS12*	40S ribosomal protein 12	12.	*ND*
DQ822665	*HwRPS15*	40S ribosomal protein 15	12. 14. 20.	*ND*
DQ822667	***HwRPS16A***	40S ribosomal protein 16A	12.	**7.6**
DQ822663	*HwRPS17A*	40S ribosomal protein 17A	12.	*ND*
DQ822668	*HwRPS26B*	40S ribosomal protein 26B	12.	*ND*
DQ822659	*HwRPS8A*	40S ribosomal protein 8A	12.	*ND*
DQ822662	*HwRPS8B*	40S ribosomal protein 8B	12.	*ND*
DQ822608	***HwSAM2***	S-adenosylmethionine synthetase	1.	**2.8**
DQ822633	***HwSHY1***	Mitochondrial inner membrane protein chaperone	2. 16. 32.	**2.5**
DQ858161	***HwSKN1***	Beta-1,6-glucan synthetase	1. 2. 42.	**2.8**
DQ822611	***HwSSA4***	Heat shock protein 70	14. 16. 32.	**1.8**
AY731090	***HwSTT3***	Catalytic subunit of the oligosaccharyltransferase complex	1. 14.	**2.8**
DQ822640	***HwSUN4***	Cell wall synthesis protein related to glucanases	1. 40. 42.	**-2.5**
DQ822676	***HwTDH1***	Glyceraldehyde-3-phosphate dehydrogenase	1. 2.	**8.9**
DQ858159	***HwTEF1***	Eukaryotic elongation factor 1-alpha (EF-1A)	12.	**2.1**
DQ822658	***HwTIF1***	Ekcaryotic translation initiation factor 1-alpha (eIF-4A)	12.	**5.0**
DQ822609	***HwTKL1***	Transketolase	1. 2.	**5.2**
DQ822632	***HwTOM40***	Mitochondrial import receptor translocase	14. 20.	**4.0**
DQ822644	*HwTPM1*	Tropomyosin	16. 20. 34. 40. 42. 43.	*ND*
DQ822647	*HwTUB1*	Alpha-tubulin	10. 14. 42.	*ND*
DQ822648	***HwTUB2***	Beta-tubulin	10. 14. 42.	**-1.8**
DQ822653	***HwUGP1***	UDP-glucose pyrophosphorylase	1. 2. 14.	**2.8**
DQ822637	*HwVPS34*	Phosphatidylinositol 3-kinase homolog	1. 14. 20. 30.	*ND*
DQ858157	*SOL10*	Weakly similar to *H. sapiens *cyclin-dependent kinase 6	*ND*	*ND*
DQ822616	***SOL11***	Mannose-P-dolichol utilization defect 1 protein	(14. 16. 20.)	**-1.5**
DQ822618	*SOL12*	Ubiquitin associated protein 2-like protein	(14. 16. 32.)	*ND*
DQ822636	***SOL13***	Opsin 1	(2. 20.)	**2.1**
DQ822638	*SOL14*	Histidine-rich glycoprotein precursor	(32. 40.)	*ND*
DQ822639	***SOL15***	Acetyl xylan esterase	(1. 32. 34.)	**2.2**
DQ822650	***SOL16***	Senescence-associated protein	(10. 11. 40.)	**1.8**
DQ822652	***SOL17***	Feruloyl esterase	(1. 32. 34.)	**2.0**
DQ822674	***SOL18***	Hyperosmolarity-induced mRNA 18	*ND*	**6.9**
DQ822675	***SOL19***	Weakly similar to yeast stress response transcription factor Crz1	*ND*	**-1.9**
DQ822676	***SOL20***	Weakly similar to yeast nitrosoguanidine resistance protein Sng1	*ND*	**2.4**
DQ822677	***SOL21***	Weakly similar to yeast prespliceosomal RNA helicase Prp5	*ND*	**7.2**
DQ822678	*SOL22*	Hyperosmolarity-induced mRNA 22	*ND*	*ND*
DQ822679	***SOL23***	Weakly similar to yeast Hsp70 protein Ssz1	*ND*	**5.5**
DQ822680	***SOL24***	Weakly similar to yeast t-SNARE protein Sso1	*ND*	**3.8**
DQ822681	***SOL25***	Hyperosmolarity-induced mRNA 25	*ND*	**5.5**
DQ822682	***SOL26***	Weakly similar to yeast transcriptional repressor Rgm1	*ND*	**6.7**
DQ858160	***SOL27***	Hyperosmolarity-induced mRNA 27	*ND*	**6.3**
DQ822683	***SOL28***	Hyperosmolarity-induced mRNA 28	*ND*	**5.0**
DQ858162	*SOL29*	Weakly similar to yeast histone deacetylase complex subunit Pho23	*ND*	*ND*

**Table 2:** Ten most frequent cDNAs in the Hw4.5-3 subtracted library. GenBank homologies, hit numbers and percentages of the most redundant cDNA clones are presented. ^a^GenBank accession number of the most similar sequence identified by BLASTX alignment. ^b^Number of clones from Hw4.5-3 subtracted library assigned to the same GenBank accession number.

BLAST hit Acc. No.^a^	Putative identity	Number of hits ^b^	Percentage (%)
AAK94755	Plasma membrane proton-exporting ATPase	13	7.6
XP750490	Heat shock protein 70	9	5.3
CAB38220	Cytochrome c oxidase subunit I	7	4.1
XP658662	Pathogenesis-related protein precursor	6	3.5
AAP57757	Acetyl xylan esterase	5	2.9
EAS31689	Unsaturated phospholipid methyltransferase	5	2.9
XP957152	Cobalamin-independent methionine synthase	5	2.9
XP660489	High mobility group protein A	4	2.4
CAA72382	14-3-3 protein	4	2.4
BAB33421	Senescence-associated protein	3	1.8
Sum of top ten redundant clones		60	35.8

**Table 3:** Distribution of differentially expressed genes from the Hw4.5-3 library by functional categories. Functional categorization was performed according to the MIPS database [22, 23] and annotated by the FunCat functional annotation scheme for systematic classification [22, 23]. Grouping of genes into indicated groups is shown as percent (%) of total identified EST from Hw4.5-3 library in descending order of abundance.

Functional category	Description	% N = 95
1	Metabolism	31.6
2	Energy	23.3
12	Protein synthesis	21.1
20	Cellular transport, transport facilitation and transport routes	20.0
10	Cell-cycle and DNA processing	17.8
16	Protein with binding function or cofactor requirement (structural or catalytic)	16.7
14	Protein fate	14.4
34	Interaction with the cellular environment	13.3
42	Biogenesis of cellular components	12.2
40	Cell fate	11.1
32	Cell rescue, defense and virulence	11.1
11	Transcription	10.0
43	Cell type differentiation	6.7
30	Cellular communication/signal transduction mechanism	4.4
41	Development (systemic)	1.1
ND	Not determined/Unknown function	13.5

### The majority of osmoresponsive genes in halotolerant *H. werneckii *are up-regulated in 4.5 M NaCl

**Figure 1:**
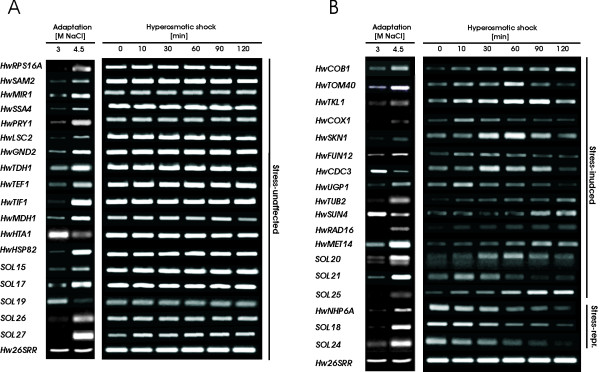
**Expression profiles of HwHog-ChIP negative genes in adapted and stressed *H. werneckii *cells**. RT-PCR was performed with RNA isolated from cells adapted to 3 M and 4.5 M NaCl (Adaptation) or cells exposed to hypersaline stress at indicated time points (Hyperosmotic stress). 26S rRNA (*Hw26SRR*) was used as an internal control for template normalization. Genes whose expression was not affected by salt-stress are presented in (A) and those that responded to salt stress are presented in (B).

**Figure 2:**
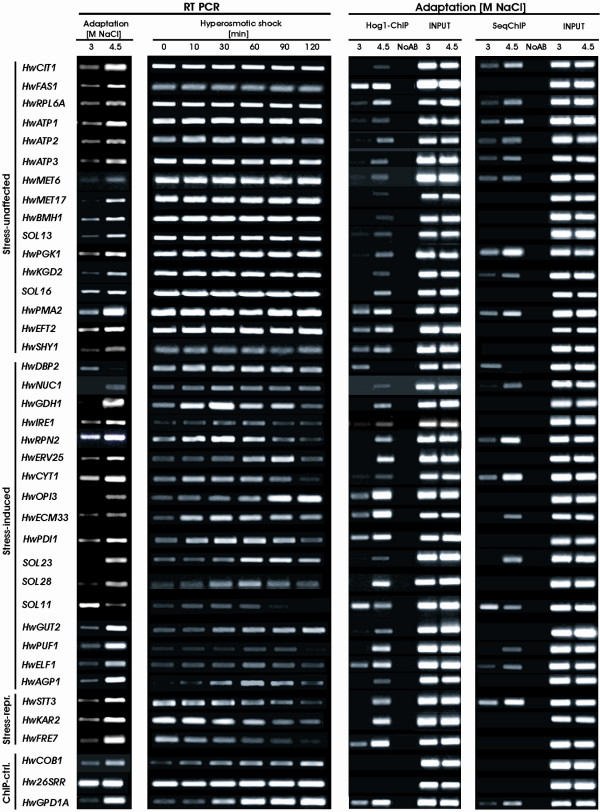
**Expression profiles of HwHog-ChIP positive genes in adapted and stressed *H. werneckii *cells and ChIP results in adapted cells**. RT-PCR was performed with RNA isolated from adapted cells (Adaptation) or cells exposed to hypersaline stress (Hyperosmotic stress) as indicated. 26S rRNA (*Hw26SRR*) was used as an internal control for template normalization. The HwHog1-ChIP and sequential RNA polymerase II/HwHog1-ChIP (SeqChIP) results for cells adapted to 3 M NaCl and 4.5 M NaCl are presented in parallel. The negative control for nonspecific binding of antibodies in both ChIP experiments is represented in the "NoAB" line and the positive control for genomic DNA amplification from 100-fold diluted *input *samples is labeled as "INPUT". *HwCOB1 *and *26SRR *genes were negative controls and *HwGPD1A *was the positive control for HwHog1 and RNA polymerase II cross-linking.

To confirm the differential expression and expression profiles of the ESTs obtained from the Hw4.5-3 library, a relative semi-quantitative RT-PCR was carried out. A total of 72 (75.8%) ESTs of interest, together with controls, were selected for further analyses. The levels of corresponding mRNAs in cells adapted to 3 M or 4.5 M NaCl were then analyzed. All of the 72 tested genes were confirmed as truly differentially expressed in *H. werneckii *adapted either to 3 M or 4.5 M NaCl. The magnitude of induction or repression of these genes is presented in Table 1, whereas the expression profiles are presented in Figures 1A, B and 2 (first columns). Fifty (69.4%) of the 72 genes were up-regulated 2- to 7-fold in 4.5 M NaCl. Besides *HwGPD1A*, used as a control (10.3-fold up-regulated), 10 genes (13.9%) were up-regulated more than 7-fold in 4.5 M NaCl compared with 3 M NaCl: *HwELF1*, *HwERV25*, *HwFRE7*, *HwGDH1*, *HwOPI3*, *HwPMA2*, *HwPRY1*, *HwRPS16A*, *HwTDH1 *and *SOL21*. The expression of these genes was virtually undetectable or barely detectable in cells from the 3 M NaCl medium. By contrast, only 6 (8.3%) of the genes studied by RT-PCR were preferentially induced in 3 M NaCl: *HwCDC3*, *HwHTA1*, *HwSUN4*, *HwTUB2*, *SOL11 *and *SOL19*. They were 1.5- to 3.3-fold down-regulated in 4.5 M NaCl. Of the 10 novel hyperosmolarity-induced *SOL *genes studied by RT-PCR, only *SOL19 *was down-regulated.

### Expression of osmoresponsive genes is different in salt-adapted and salt-stressed *H. werneckii *cells

Induction of gene transcription has been recognized as a crucial mechanism for the adaptation of yeast cells to saline stress. Accurate whole genome expression analysis by microarray has been performed on *S. cerevisiae *at different salt concentrations (0.4 M–1.0 M NaCl) and different time points (10–90 min), resulting in different patterns of gene expression [2,25]. We asked if the salt concentration-dependent differences in the expression of *H. werneckii *osmoresponsive genes are characteristics of the adaptation phase only, or if these genes would also respond to acute hypersaline stress. To study the acute stress response, *H. werneckii *cells were exposed to hypersaline stress and the level of gene expression over the next 120 minutes was followed by RT-PCR. As shown in Figures 1A, B and 2 (second columns), time-dependent gene expression was observed only for half of the genes under study. Genes were nominated as "salt-stress responsive" if the mRNA level was induced or repressed at least 2-fold during the 120 min period following the salt stress. Whereas 38 (52.8%) genes were shown to be responsive to the acute hypersaline stress (see Table 4 for the list of the stress-responsive genes), the expression of 34 (47.3%) genes was not susceptible to the stress. As salt-stress responsive genes responded with different time courses of induction or repression, they were grouped into 21 early-induced genes and 11 delayed-induced genes if the 2-fold increase in mRNA level was observed within or after the first 30 min of salt stress, respectively; and 6 repressed genes, whose of mRNA level decreased within the first 10–30 min after salt stress. Those genes that were induced more than 5-fold during salt stress were *HwECM33*, *HwRAD16*, *HwRPN2*, *HwSKN1*, *HwUGP1 *and *HwGPD1A *as a control from the early-induced genes; and *HwOPI3*, *HwSUN4*, *SOL23 *and *SOL28 *from delayed-induced genes. RT-PCR examination of *SOL20 *repeatedly resulted in two PCR products (Figure 2B), suggesting a co-amplification of similar genes, both up-regulated in adapted cells (4.5 M NaCl), whereas it seems that only one of them responded to hypersaline stress. Among repressed genes,*HwFRE7*, *HwNHP6A *and *SOL24 *were more than 5-fold down-regulated after acute salt stress.

**Table 4:** Induced and repressed genes in *H. werneckii *upon acute hypersaline stress. The stress-responsive genes were classified according to the kinetic of the response over the 120 min course of the experiment. Fold induction or repression is indicated as mean value of three independent RT-PCR experiments, and the representative gels are shown in figures 1 and 2.

**EARLY induced**		**DELAYED induced**		**REPRESSED**	
Gene	Fold induction	Gene	Fold induction	Gene	Fold repression
*HwAGP1*	5.2	*HwELF1*	2.5	*HwFRE7*	7.0
*HwCDC3*	4.7	*HwERV25*	3.0	*HwKAR2*	4.4
*HwCOB1*	3.1	*HwGUT2*	4.0	*HwNHP6A*	5.2
*HwCOX1*	2.8	*HwMET14*	3.1	*HwSTT3*	3.6
*HwCYT1*	8.3	*HwOPI3*	6.2	*SOL18*	3.3
*HwECM33*	5.5	*HwPUF1*	2.7	*SOL24*	5.5
*HwFUN12*	2.4	*HwSUN4*	5.7		
*HwGDH1*	4.2	*HwTOM40*	3.4		
*HwGPD1A*	6.3	*SOL23*	5.3		
*HwIRE1*	4.7	*SOL25*	4.4		
*HwNUC1*	3.0	*SOL28*	5.6		
*HwPDI1*	3.9				
*HwRAD16*	7.0				
*HwRPN2*	5.6				
*HwSKN1*	5.8				
*HwTKL1*	3.9				
*HwTUB2*	2.6				
*HwUGP1*	7.5				
*SOL11*	3.2				
*SOL20*	2.8				
*SOL21*	3.1				

These data demonstrate that in contrast to the response of the adapted cells, only a portion of the identified differentially expressed genes in *H. werneckii *transcriptionally responded to acute hypersaline stress as well. Interestingly, *HwFRE7, HwKAR2, HwNHP6A, HwSTT3, SOL18 *and *SOL24 *were up-regulated in cells adapted to extreme hypersaline conditions, but they were actually repressed after acute hypersaline stress. Additionally, *HwCDC3, HwSUN4 *and *SOL11 *were down-regulated in cells adapted to extreme hypersaline conditions, but induced during stress. This observation indicates an additional level of complexity in regulation of gene expression in halotolerant yeast when compared with the stress response of salt-sensitive *S. cerevisiae*.

It is noteworthy that the expression profile of *H. werneckii *adapted to 4.5 M NaCl is very different from the hypersaline stressed cells of *S. cerevisiae*. In comparison with the microarray studies performed with *S. cerevisiae *[2,25,26], using SSH-MOS we have identified only 18 gene orthologs that were clearly up-regulated in adapted or stressed *H. werneckii *and also in salt-stressed wild-type *S. cerevisiae*: *HwBMH1*, *HwCIT1*, *HwGND2*, *HwGUT2*, *HwHSP82*, *HwKGD2*, *HwMDH1*, *HwOPI3*, *HwRPL16A*, *HwRPS10B*, *HwRPS15*, *HwRPS8A*, *HwSHY1*, *HwSSA4*, *HwTDH1*, *HwTIF1*, *HwTKL1*, and *HwUGP1*. However, some discrepancies were observed between the compared microarray data in *S. cerevisiae *studies mentioned above. Among upregulated genes in salt-stressed *S. cerevisiae*, only *HwGUT2*, *HwOPI3*, *HwTKL1 *and *HwUGP1 *were also salt-stress responsive in *H. werneckii*. The mRNA levels of *HwECM33*, *HwEFT2*, *HwFAS1*, *HwFUN12*, *HwGDH1*, *HwMET14*, *HwMET17*, *HwMET6*, *HwMIR1*, *HwNUC1*, *HwPMA2*, *HwPRY1 HwRPL6A*, *HwRPS16A*, *HwSAM2*, *HwSTT3*, *HwTEF1*, *HwTIF1 and HwTOM40 *were induced in *H. werneckii *adapted to 4.5 M NaCl, whereas these levels diminished or remained unaffected in salt-stressed *S. cerevisiae*. Two genes, *DBP2 *and *SUN4 *were substantially downregulated in both, the hypersalinity-adapted *H. werneckii *and salt stressed *S. cerevisiae*. It was also found that *GND2*, *GPD1 *and *SSA4 *belong to a common environmental response (CER) genes in *S. cerevisiae*, which were affected not only by high salinity but various stresses such as heat, high or low pH, oxidative stress and sorbitol [27] and therefore might also present the CER response in *H. werneckii*. Interestingly, the induction of *SSA4 *was extremely stress-responsive in *S. cerevisiae *(47-fold induction), whereas it was only slightly affected by high salt concentration in *H. werneckii *(1.8-fold in 4.5 M NaCl).

One theory is that the main difference between salt-sensitive and halotolerant organisms in the expression of osmoresponsive genes relates to the inducibility and/or maintenance of the transcription level of protective genes. The perception threshold for the extracellular "hyper"-osmolarity in extremely halotolerant yeasts must be set at concentrations higher than salt-sensitive unicellular eukaryotes could even survive. In *H. werneckii *adapted to these extreme conditions, the protective response remains "on", meaning that there is a long-term up-regulation program of specific genes, and this up-regulation does not decrease over time.

### HwHog1 associates with 36 novel osmoresponsive genes in chromatin of long-term adapted cells

The transcriptional induction or repression of approximately 500 genes in *S. cerevisiae *that are strongly responsive to salt stress was highly or fully dependent on the hyperosmolarity-responsive MAPK Hog1, indicating that the Hog1-mediated signaling pathway plays a key role in global gene regulation under saline stress conditions [2,28]. It has been shown that following exposure to salt stress, Hog1 is retained in the nucleus and becomes associated with the chromatin of target genes [12]. We approached the study of endogenous HwHog1 interaction with the chromatin regions of identified up-regulated genes in adapted *H. werneckii *cells by a chromatin immunoprecipitation (ChIP) PCR assay. Lacking information on promoter regions for the identified differentially-expressed genes in *H. werneckii*, a ChIP-coding region PCR amplification was performed. It has recently been shown that the activated Hog1 in *S. cerevisiae *is associated with elongating RNA polymerase II and is therefore recruited to the entire coding region of osmoinducible genes [29]. *HwGPD1A *was used as a positive control for the HwHog1-ChIP. As negative controls for the association of HwHog1 with DNA, the *HwCOB1 *gene encoded in mitochondrial DNA and the 26S rRNA gene (*Hw26SRR*) transcribed by RNA polymerase I were used.

The results of the HwHog1-DNA interactions determined by ChIP are shown in Figure 2, third column. As evidenced by PCR, the protein HwHog1 cross-linked with the coding region of the positive control *HwGPD1A *and 36 of the differentially expressed genes (50%), but not with the negative control genes *HwCOB1 *and *Hw26SRR*. As seen from the PCR product level, for 34 up-regulated genes the interaction with HwHog1 was stronger in cells adapted to 4.5 M NaCl. In contrast, for 2 down-regulated genes (*HwDBP2 *and *SOL11*), the HwHog1-ChIP signal was stronger in cells adapted to 3 M NaCl.

Genome-wide expression profiling studies using wild-type and *hog1 *mutant *S. cerevisiae *cells were performed to comparatively identify genes whose up-regulation of expression was dependent on Hog1. Of several hundred genes whose RNA levels were Hog1-dependent, a relatively small subset of approximately 40 high-osmolarity induced genes had a strong requirement for Hog1 for their induction [26,28]. Among them, only the *UGP1 *ortholog was induced in 4.5 M NaCl adapted and salt-stressed *H. werneckii *cells. Other yeast orthologs of HwHog1-ChIP positive genes in *H. werneckii *were reported for the first time in the present study in connection with MAPK Hog1. However, the HwHog1-ChIP did not confirm the HwHog1 interaction with the *HwUGP1 *gene in salt-adapted *H. werneckii*. The relative distribution of HwHog1-dependent genes was approximately equivalent among functional categories, except in the case of transcription, cellular transport, signal transduction mechanism and cell fate categories (MIPS categories 11, 20, 30 and 41, respectively), where the HwHog1-ChIP positive genes represented more than 70% of tested genes. Only 2 of 10 tested *SOL *genes (*SOL23 *and *SOL28*) with unknown functions (*ND *in table 1) were HwHog1-ChIP positive. Caution must be applied to the interpretation of genes which were HwHog1-ChIP negative, since many of them were salt-stress responsive. These genes could still be regulated by HwHog1. It is possible that during long-term adaptation the continuous interaction of HwHog1 with their genomic region is not obligatory, and thus HwHog1 was not cross-linked with the chromatin. Alternatively, HwHog1 could activate responsible factors more distal to the chromatin, thereby avoiding the cross-linking range.

### HwHog1 and RNA polymerase II co-localization in coding regions of osmoresponsive genes is reflected by elevated levels of corresponding transcript in adapted cells

It has been previously shown that in the HOG response, the nuclear retention and chromatin association of Hog1 in *S. cerevisiae *depends on co-localization with general transcription machinery components [11,12]. We further asked whether HwHog1 cross-linking occurs with co-localization of RNA polymerase II in the case of HwHog1-dependent genes. A sequential HwHog1-ChIP analysis (SeqChIP) using primers specific for the genes identified as HwHog1-positive was therefore performed after the primary RNAPolII-ChIP. If the interaction of HwHog1 and RNAPolII existed within the same genomic region of HwHog1-positive genes, the PCR signal should be obtained in eluates of SeqChIP. As shown in Figure 2, fourth column, the co-localization of HwHog1 and RNA polymerase II existed in 17 cases out of 36 HwHog1-ChiP positive differentially expressed genes. Similarly, if the co-occupancy of HwHog1 and RNAPolII favored gene expression, then the relative ratio of amplified PCR products from SeqChIP eluates should reflect the relative ratio of RT-PCR results from the gene expression in adapted cells. As shown in Figure 2, the ratio of amplified PCR products in SeqChIP-positive genes does indeed reflect the ratio of mRNA levels observed by RT-PCR in both conditions of adaptation. Co-occupation of HwHog1 and RNA polymerase II in target genes resulted in an increased PCR signal in SeqChIP, with an accompanying increased level of corresponding transcript in RT-PCR analyses.

Taken together, these observations indicate a stimulating role for HwHog1 and RNA polymerase II co-localization on the efficiency of transcription of indicated genes, even in long-term high-salt adapted cells. Regarding the coding region positioning of primer pairs used in this study, we can say that in *H. werneckii *HwHog1 also associates with the elongating RNA polymerase II, as has recently been shown in *S. cerevisiae *[29]. In our study, *H. werneckii *cells used for chromatin immunoprecipitation analyses were completely adapted to extremely high environmental salt concentration by long-term growth in media containing either 3 M or 4.5 M NaCl. These results thus reflect HwHog1/RNAPolII-chromatin interactions, relevant for the extremely high saline conditions which until now could not be studied in salt-sensitive organisms. Moreover, to date this study is the first large-scale exploration of Hog1 interaction with target genes by chromatin immunoprecipitation in an organism with unavailable genomes. All similar studies have been performed using tagged and over-expressed proteins. Our study relays information based entirely on the cross-linking of endogenous HwHog1 and RNA polymerase II with their downstream targets on the chromatin, showing actual physiological interactions never studied before in eukaryotic cells adapted to such an extremely hyperosmotic condition.

### An integrative model of osmoresponsive gene action through functional modules in *H. werneckii*

**Figure 3:**
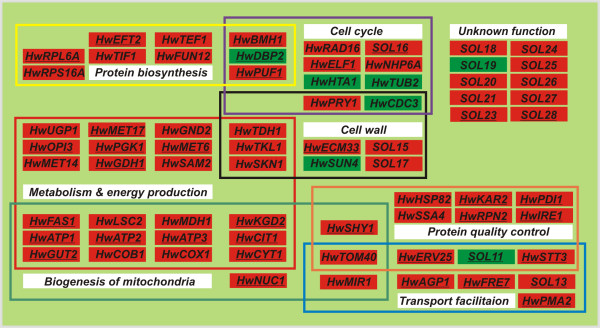
**Model of gene interactions through functional modules responsible for the adaptive metabolism of *H. werneckii *in an extremely hypersaline environment**. Interactions between functional modules are presented by overlapping frames. For visualization purposes, up-regulated genes are presented in red boxes and down-regulated genes are in green boxes. HwHog1-ChIP positive genes are underlined.

The transcriptional behavior of some genes from our analysis correlated with known biochemical hyperosmotic stress responses in *S. cerevisiae *(for review see [4]). Nevertheless, important differences were observed between the two organisms, which presumably contribute to the remarkable capacity of *H. werneckii *for adaptation to extreme environments. We propose the following model of gene action through functional modules in *H. werneckii *adaptation to a hypersaline environment (Figure 3). During long-term adaptive growth in extreme salinity (4.5 M NaCl), there is a constant need to pump protons across the plasma membrane, which provides the electrochemical driving force for the Na^+^/H^+^-antiporters and P-type ATPases *HwENA1/2 *to exclude the intruding sodium cations [30] and to maintain the correct intracellular pH and plasma membrane potential. The 7.9-fold up-regulation of proton-exporting plasma membrane ATPase gene *HwPMA2*, which is also represented as the most frequent EST from the Hw4.5-3 subtracted library, indicates the importance of cytoplasmic pH regulation and proton gradient across the plasma membrane. We have observed a substantial decrease in growth media pH from pH 7.5 down to pH 2.5 during the log phase, which was more evident and faster with *H. werneckii *growing in 4.5 M NaCl than in 3 M NaCl (unpublished data). Therefore the cell has to increase its energy production machinery to supply the energetic demands of ion homeostasis. The functional category of energy supply was highly represented among up-regulated genes (Tables 1 and 3) in 4.5 M NaCl, including those coding for components of the electron transport chain (*HwCOB1*, *HwCYT1*, *HwCOX1*) and ATP production (*HwATP1*, *HwATP2*, *HwATP3*), and conveying the excessive cytosolic NADH into the mitochondrial respiratory chain (FAD-dependent glycerol-3-phospate dehydrogenase, *HwGUT2*). More than 10-fold up-regulation of *HwFRE7 *gene could indicate the increased requirements for the ferric-chelate reductase activity – namely, to satisfy the need for enhanced iron assimilation or reduction of oxidized iron-containing complexes under extremely hypersaline condition. Energy production metabolism seems to be additionally enhanced by the up-regulation of genes coding for the enzymes of the glycolytic pathway (*HwTDH1*, *HwPGK1*), the tricarboxylic acid cycle (*HwCIT1*, *HwKGD2*, *HwLSC2*, *HwMDH1*, *HwGDH1*), the pentose phosphate pathway (*HwTKL1*, *HwGND2*), and modulation of energy storage (*HwUGP1*, *HwGAL10*). More than 8-fold up-regulation of a specific mitochondrial inner-membrane Pi transporter gene, *HwMIR1*, further supported the idea of increased energy demands in *H. werneckii *growing at 4.5 M NaCl as compared to cells growing in a lower salinity, since most cellular ATP is produced within the mitochondria from ADP and Pi. Many of the Hw4.5-3 genes described so far, together with *HwSUN4*, *HwTOM40*, *HwSHY1 *and *HwNUC1*, code for mitochondrial enzymes, indicating the process of continuous adaptation to hypersaline conditions must be strongly supported by the biogenesis of mitochondria. Indeed, in the subcellular fractionation experiment we found that the quantity of isolated mitochondria was at least 2-fold higher in cells from 4.5 M NaCl, and that the succinate dehydrogenase activity per μg of mitochondria was 2.5 ± 0.5-fold higher compared with mitochondria from 3 M NaCl (data not shown). The biogenesis of mitochondria is coupled with elevated protein synthesis. Specific ribosomal protein isoforms (Table 1) and translation-regulating factors (*HwFUN12*, *HwTIF1*, *HwEFT2*, *HwTEF1*) are synthesized, which could be more effective in the translation of desired transcripts under unfavorable conditions and thereby increase the proliferative potential of the cell. The quantity of synthesized or damaged proteins could impair the ability of the cell to properly fold the resultant proteins; thus the components of protein quality control and the unfolded protein response (*HwHSP82*, *HwSSA4*, *HwERV25*, *HwRPN2*, *HwSTT3*, *HwIRE1, HwKAR2*) are constantly up-regulated. The increased protein synthesis is also coupled with up-regulation of genes coding for enzymes from amino acid biosynthetic pathways (*HwMET6*, *HwMET14*, *HwMET17*, *HwSAM2*, *HwGDH1*) and an amino acid permease (*HwAGP1*). From this point of view, the basic metabolic network is constitutively working to meet the demands of the increased energy expenditure needed to balance the ionic and the osmotic homeostasis. Because the environmental osmolarity of the natural habitat of *H. werneckii *can change, the plasma membrane has to be fluid enough, enabling the proper dynamics of signaling processes in a wide range of environmental salinities. Electron paramagnetic resonance spectroscopy measurements showed that the membranes of all halophilic fungi in extremely saline environments were more fluid than those of the salt-sensitive *S. cerevisiae*, which was in close agreement with the sterol-to-phospholipids ratio and fatty acid unsaturation level [19]. Plasma membrane fluidity could be supported by the biosynthesis of unsaturated or methylated lipids as a consequence of the 9.7-fold up-regulation of *HwOPI3 *and related genes (*HwFAS1*, *HwSKN1*). The interaction of cells with the cellular environment is mediated through the cell wall, which is highly implicated in the regulation of cellular turgor pressure. Two poorly described *S. cerevisiae *orthologs connected to cell wall integrity and architecture encoded by *HwECM33 *and *HwSUN4 *were identified as salt-responsive in *H. werneckii*. The finding that the major component of the septin complex *HwCDC3 *was strongly down-regulated, together with *HwSUN4*, could explain the substantially prolonged generation period of *H. werneckii *cells grown in 4.5 M NaCl compared to those grown in 3 M NaCl [15]. The role of interaction with the cellular environment is also assigned to *SOL15 *and *SOL17*, coding for acetyl-xylan esterase and feruloyl esterase orthologs, respectively, and to one of the most up-regulated genes at 4.5 M NaCl, the *HwPRY1 *gene, putatively coding for pathogenesis-related proteins in plants. The roles of both esterases in the automodulation of the cell wall dynamic as an adaptive response to high turgor pressure remains speculative.

## Conclusion

In our study, we have identified a set of 95 osmoresponsive genes in the extremely halotolerant black yeast *H. werneckii *adapted to a moderately saline environment of 3 M NaCl or an extremely saline environment of 4.5 M NaCl. Among them, more than half were related with general metabolism and energy. Thirteen unclassified *SOL *genes represent a specific transcriptional response unique to *H. werneckii*. A novel offset of 36 genes was shown as Hog1-dependent in long-term adaptation to extreme environments, previously not assigned as such in the salt-sensitive model organism *S. cerevisiae*. The combined data indicate important differences in the cellular processes of osmoadaptation between halotolerant and salt-sensitive yeasts. The novel set of osmoresponsive genes probably represents only a portion of actual differentially expressed genes in *H. werneckii*; however, we believe that valuable information was obtained concerning genes related to the hypersaline adaptation of extremely halotolerant eukaryotes.

## Methods

### Cell growth conditions

The *H. werneckii *strain MZKI B736 was obtained from culture collections of the Slovenian National Institute of Chemistry. Cells were grown at 28°C and 180 rpm in the defined YNB medium (0.17% (w/v) yeast nitrogen base, 0.08% (w/v) complete supplement mixture, 0.5% (w/v) ammonium sulphate, 2% (w/v) glucose in deionized water, pH 7.0), supplemented with the indicated NaCl concentration. Cells were harvested in the mid-exponential phase (OD_600 nm _0.6–0.8) and frozen in liquid nitrogen. For hypersaline stress, *H. werneckii *cells were grown in YNB with 1 M NaCl to OD_600 nm _0.8 and then transferred to the medium containing 4.5 M NaCl. Aliquots of the culture were removed before the stress was induced, and then at 10, 30, 60, 90 and 120 min after the stress. Cells were separated from the growth medium by fast filtration through a 0.45 μm-pore filters and then frozen in liquid nitrogen.

### Suppression subtractive hybridization, mirror orientation selection and differential screening of subtracted library

Total RNA from cells of *H. werneckii *was isolated using TRI Reagent (Sigma-Aldrich) according to the manufacturer's instructions from mid-exponential phase cells grown in YNB media with 3 M or 4.5 M NaCl. The poly(A)RNA was isolated using the Oligotex mRNA Mini Kit (Qiagen). 1 μg of poly(A)RNA from each sample was used for reverse transcription to perform the cDNA suppression subtractive hybridization using the Clontech PCR-select cDNA Subtraction Kit (BD Bioscience) and Advantage cDNA PCR Polymerase Mix (BD Bioscience) according to the manufacturer's protocols. cDNA from a sample of cells growing in YNB medium with 4.5 M NaCl was used as a tester, while a sample from the 3 M NaCl medium was used as a driver in forward subtraction (and *vice versa *for reverse subtraction). Mirror orientation selection was implemented as described by Rebrikov *et. al*. [20] with some modifications. Briefly, after subtractive hybridization, the primary PCR of 27 cycles was performed in 5 parallel tubes. The samples were then combined, diluted 500-fold and subsequently amplified by 12 additional PCR cycles using the same primer and conditions as described for the primary PCR. A secondary PCR was then performed as described in the manufacturer's protocol. 150 ng of the resultant subtracted cDNA samples were digested with 10 U of *Cfr9*I (Fermentas) for 1 h at 37°C in total volume of 20 μL. 1 μL of *Cfr9*I-digested cDNA was mixed with 1 μL of 4 × hybridization buffer (2 M NaCl, 200 mM HEPES pH 8.3, 0.8 mM EDTA), 2 μL of water, denatured at 98°C for 1.5 min, and then hybridized at 68°C for 4 h. Samples were then diluted with 200 μL of the dilution buffer (50 mM NaCl, 20 mM HEPES pH 8.3, 0.2 mM EDTA) and heated at 70°C for 7 min. 1 μL of diluted cDNA was taken for 20 μL tertiary PCR with 0.6 μM adapter-specific primer NP2Rs (5'-GGTCGCGGCCGAGGT-3') by the following temperature program: 2 min at 72°C for initial extension of 3'-ends, followed by 23 cycles with 7 s at 95°C, 20 s at 62°C, and 2 min at 72°C. 1.5 μL of PCR products was cloned into pGEM-T Easy Vector (Promega) and transformed in JM109 competent cells (Promega). After blue/white selection on LB-Ampicillin/IPTG/X-Gal plates, white colonies were picked and arrayed on LB-Ampicillin plates, including 2 blue colonies as negative hybridization controls for the differential screening. Two identical colony lifts were made from each plate onto nitrocellulose membranes (Sigma), soaked with denaturation solution (0.5 M NaOH, 1.5 M NaCl), neutralizing solution (1.5 M NaCl, 0.5 M Tris-HCl, pH 7.4) and finally with washing solution (2 × SSC, 0.5 M Tris-HCl, pH 7.4), and then fixed by baking for 1–2 hours at 80°C. Forward and reverse subtracted hybridization probes were prepared from SSH-MOS secondary PCR products digested with *Rsa*I, *Cfr*9I and *Eae*I to remove and degrade adaptors. After clean-up, 100 ng of each probe was labeled with [γ-^32^P]dCTP. Colony lifts were pre-hybridized with hybridization solution (5 × SSC, 0.5% SDS, 5 × Denhard's reagent, 0.1% SDS, 150 μg/mL salmon sperm DNA) for 1 h at 72°C, and then hybridized overnight at 72°C with labeled probes. Membranes were then washed once with low-stringency solution (2 × SSC, 0.5% SDS), twice with high-stringency solution (0.2 × SSC, 0.5% SDS), each for 20 min at 68°C, and then exposed to BioMax MR film (Kodak) overnight. For the forward-subtracted cDNA library (Hw4.5-3), clones that hybridized only to the forward-subtracted probe but not to the reverse-subtracted probe and clones that hybridized to both subtracted probes with a difference in signal intensity >5-fold were assigned as truly differential and chosen for sequencing. Plasmid DNA from positive clones was isolated using the Wizard Plus Minipreps Purification System (Promega), sequenced and analyzed by BLAST algorithms.

### Reverse transcription PCR

The total RNA was isolated as described above and treated with DNase I (Fermentas). 1 μg of RNA was used for 20 μl of reverse transcription reaction using Superscript III Reverse Transcriptase (Invitrogen, USA) and random hexamer-primers (Promega) according to the manufacturer's protocols. PCR with Gotaq DNA polymerase (Promega) was performed using 0.5 μl of cDNA in a 20 μl PCR reaction with 15 nmol of specific primers listed in Additional file 1. Thermal cycling was programmed for 23 cycles, each consisting of 30 sec at 94°C, 30 sec at 60°C and 30 sec at 72°C. The cycle number of 23 was empirically determined in the optimal linear range of amplification by measuring the concentration of PCR products after the 20^th^, 22^nd ^and 24^th ^cycles. PCR products were resolved in agarose gels, documented by MiniBis (DNR BioImaging Systems), and the band intensities quantified with the TotalLab gel analysis program (Nonlinear Dynamics).

### Chromatin immunoprecipitation

Immunoprecipitation of cross-linked chromatin (ChIP) was performed as described by Hecht *et al.*[31] and Proft *et al.*[32], and sequential ChIP (SeqChIP) as described by Geisberg *et al*. [33] with some modifications. Briefly, cells of *H. werneckii *growing in YNB media with 3 M or 4.5 M NaCl were cross-linked at OD_600 _0.8 using formaldehyde in a final concentration of 1% for 15 min at room temperature. Cross-linking was stopped with glycin at a final concentration of 0.125 M for 5 min at room temperature. Cells were harvested, washed twice with ice-cold PBS, pelleted, frozen in liquid nitrogen and broken with a dismembrator. 2.5 g of powdered cells were re-suspended in 10 mL of ChIP-L buffer (1% SDS, 10 mM EDTA, 50 mM Tris-HCl, pH 8.1) containing a cocktail of fungal protease inhibitors (Sigma) and mixed by inversion for 30 min on ice. The samples were sonicated for five 15-s pulses, resulting in DNA fragments with an average length of 500 bp, and then centrifuged for 15 min at 10.000 × g to remove insoluble debris. For each ChIP experiment, 100 μL of supernatant was frozen as an *input *and 1 mL of supernatant was diluted in 9 mL of ChIP-D buffer (0.01% SDS, 1.1% Triton X-100, 1.2 mM EDTA, 16.7 mM Tris-HCl, pH 8.1, 167 mM NaCl) with inhibitors. Chromatin solutions were pre-cleared with the salmon sperm DNA-precoated protein G-sepharose (Amersham) and then incubated with 5 μg of rabbit anti-Hog1 antibody (Santacruz) or mouse monoclonal anti-RNAPol II antibody 4H8 (Abcam) overnight at 8°C. 250 μL of precoated protein G-sepharose was added to each sample. Immunocomplexes were precipitated for 2 h at 8°C with shaking, pelleted 1 min at 1000 × g, and then pellets were washed with 2 mL of the following washing buffers: twice with ChIP-W1 (150 mM NaCl, 0.1% SDS, 1% Triton X-100, 2 mM EDTA, 20 mM Tris-HCl, pH 8.1), once with ChIP-W2 (500 mM NaCl, 0.1% SDS, 1% Triton X-100, 2 mM EDTA, 20 mM Tris-HCl, pH 8.1), once with ChIP-W3 (250 mM LiCl, 1% Na-deoxycholate, 1 mM EDTA, pH 8.0) and twice with ChIP-W4 (10 mM Tris-HCl, 1 mM EDTA, pH 8.0). Immunocomplexes were eluted twice with 200 μL of ChIP-E buffer (1% SDS, 100 mM NaHCO_3_) for 10 min at 65°C and eluates were collected. For SeqChIP, the RNAPolII-ChIP eluates (400 μL) were diluted with 3.6 mL of ChIP-dilution buffer and incubated for 2 h at room temperate with 1 μg of rabbit anti-Hog1 antibodies, and then processed as described above. ChIP eluates and *inputs *were reverse cross-linked in 0.2 M NaCl for 5 h at 65°C, incubated with 20 μg of RNase for 30 min at 37°C, followed by treatment with 10 μg of proteinase K. DNA was purified using the Wizard PCR Clean-up Purification System (Promega) and eluted with 150 μL of water for Hog-ChIP samples or with 50 μL for SeqChIP samples. PCR with Gotaq DNA polymerase was performed using 1 μl of eluted DNA from immunoprecipitated samples or 1 μl of 100-fold diluted *input *in 20 μl PCR reaction with Gotaq DNA polymerase and 15 nmol of specific primers (Additional file 1). Thermal cycling was programmed for 30 cycles, each consisting of 30 sec at 94°C, 30 sec at 55°C and 60 sec at 72°C.

## Authors' contributions

TV designed the study, carried out the molecular experiments and bioinformatics study, and drafted the manuscript. AP conceived of the study, participated in its design and coordination, and helped to draft the manuscript. Both authors read and approved the final manuscript.

## Supplementary Material

Additional file 1 List of primers used in this study. Click here for file
