# Supplementary material for: Differential gene expression and Hog1 interaction with osmoresponsive genes in the extremely halotolerant black yeast Hortaea werneckii
Source: BMC Genomics. 2007 Aug 16;8:280. doi: 10.1186/1471-2164-8-280 (PMC2034391; doi:10.1186/1471-2164-8-280)
Supplement: Additional file 1 — List of primers used in this study. [file 1471-2164-8-280-S1.pdf]

Additional file 1: Primers list. Gene names, sequences and sizes of corresponding PCR products (cDNA based) obtained with primers for RT-PCR and ChIP-PCR analyses.

**Gene name**	Forward primer [5'→3']	Reverse primer [5'→3']	Product size [bp]
Hw26SRR	CATCACTGTACTTGTTCGCTATCGGTC	GTAACGGCGAGTGAAGCGGC	279
HwAGP1	GCGGTAATGACCACGAAGTT	TCAAGGCCTTCGGTGAACTA	451
HwATP1	CTGAGGTCTCCTCCATCCTC	GAGGTACGCTTGACGGTCTC	259
HwATP2	GTCAGACCAGTCAGGGCAAC	CCGTGTTCATTCAGGAGCTT	223
HwATP3	GTTCTTGGAGGCGTTGTCC	GATCTCCCTCACCTTCACCA	343
HwBMH1	GGCCTGGTCCTCAACTTCTC	GCTCTCCTCAGACAGGGTGT	126
HwCDC3	CAACGTTATGGTCGTTGGTG	AGAGTGTCGGACTTCGCAAT	450
HwCIT1	CGTAGGCCTTGGAGAAGCTA	CCTCGACTCTGAGGAGGGTA	305
HwCOB1	AATTTGCTACAAATGCAAAAAGG	GCAGGTTCTGGAAATCCTTTAG	387
HwCOX1	TCAGGATGACCGAAAAATCA	CCGCTTTCAGGAATTCAAAG	335
HwCYT1	GTCTACCGCGAAGTCTGCTC	TCCTCGGGATAACCAGTCAG	317
HwDBP2	CTTTCCGGATCCAGTCTCAG	CGCGAAAACCAGATGACC	201
HwECM33	CCCTTAATGACACCGCTTCT	TCGGTGGTGACCTCTCTTTC	295
HwEFT2	GGAGATGGCAGGTGAAGAAT	AGCTTGAGCGTGCTTTCAAC	232
HwELF1	AGACTCCAAGCCCATCTTTG	ACAAGAAACCGACCTCATCC	247
HwERV25	AGAAGCTGAAGCCCATGGAA	CGGAAGTACGCCCTCAAATA	190
HwFAS1	CCTCCGATCCGTCAGATG	TAGAGGCATCGGAAACCTTG	224
HwFRE7	GCTTTGGCTTCCAGAATGTC	AGCTTGATTCGGAGGTTTTG	217
HwFUN12	GACTTCCTTAGCTCCCGTGAC	AAGGTTGACAAGGATGCACA	294
HwGDH1	CCTCGAAGATGTCGATAGCC	AGGAGTCCTTCAAGGGCAAG	426
HwGND2	GAAGCAGAGACGTTACCACCA	CTCCAGAAGCCGGAGATTG	401
HwGPD1A	CGAGTCCTTCACCGAGGAGTCCT	ACGGCAGGCCAAAAAGAAAAG	336
HwGUT2	CTGCCATGGAAGGAAGAAGA	CCCATGCTCAAGAAGGAGAA	435
HwHTA1	TACCTGGCTGCTGAGATCCT	GTGAATGTTGGGGAGGACAC	168
HwHSC82	TCTTGAGGGACTTGGTCAGG	AGGACAAGGAGCAGTTCGAC	441
HwIRE1	TCCGGTACTTCTGCCAGCAA	TCGTAGGGGTGGATACCATC	571
HwKAR2	AAGAAGGCCAGCAAGGGTAT	GGTGATGGTGATGGACTCG	494
HwKGD2	TCAATGCTGGCATTCACG	GATGAACCGTATGCGTCTGA	231
HwLSC2	GATCTTGAGGCCACTGTCGT	CGTCAGGAGGAGGTCTTCAG	432
HwMDH1	CGTCTCTGAGCTCGCTCTTT	GACACCCCTGGACTTGAAGA	337
HwMET14	CTTACCCGTCAGGAGCGTAG	AGTCGGTGTTCTTGGTGTCC	346
HwMET17	GACATGCCGAGAGATTGGAT	AGGCAGCGGTAGACCATCT	409
HwMET6	TCAAGGACTGGTTCGCTTTC	GGGACTGCATGCTCTTGG	126
HwMIR1	GCTGTCTGCTGTTCCATCAC	TACCTTCGTTTTGGCCATCT	422
HwNHP6A	GAGAAAGAAGGACCCCAACA	GAGCCTTCTGCTTGTCGTTC	167
HwNUC1	CGCCACCTAACGTAGCAGTT	ATCGTCTTCTGCCTCTCCTG	280
HwOPI3	GGTCAAGACGAGGATGTTGC	ACGTTCTGGAATACCGTTGC	243
HwPDI1	TGGTTGGTTTCTTCGGAAAG	TTGATCTTGCCCTTCTGCTT	388
HwPGK1	CATACTTGATCGTGGGCAGA	GCTTGCGGATACCAACACAT	188
HwPMA2	TACCGCCAACCAGCTCTCTA	CTTGGAGACAGGGTCGAAAG	223
HwPRY1	GCGTTGACCTCAGCATTGTA	CCTACGCGGACAGAGTTTTG	250
HwPUF1	GACGTAGTTGGCGAAGGAGT	CTGCAGAAGGGCAGACTTGT	300
HwRAD16	GCTCTTCTGCTGGCTTTGAT	CTCAACTTCGGTGACAAGCA	324
HwRPN2	GAGAACGAAAACGCTCAGGA	GTTGCCACGATGAATGACAC	411
HwSAM2	GCAACACCTTCCTTTTCACC	GCCTCCTCATAGTGCAGACC	334
HwSHY1	CGAGGGCAATCCAACAAC	GAATTTCCCCCTCGCGTAT	303
HwSKN1	AGCGAAACAGAGACCTCGAA	CCATTTCGACAAGGTTCAAGA	120
HwSSA4	GCAACACCTTCCTTTTCACC	GCCTCCTCATAGTGCAGACC	334
HwSTT3	TCACCGTCTCTGGCGTTG	GTCTGGCAAAGCAGAGAAC	446
HwSUN4	GCAGGTGAGGTTGCTGGT	GGCTCATACTGCTCCTACGC	294
HwTDH1	CCAGCAGTGTTGGTGAAAGA	ATCATCCTCTGCTCCGTTCC	165
HwTEF1	CAGCGTCACCAGACTTGATG	AGTGAAGTCCGTCGAGATGC	335
HwTIF1	AAGACCGCTACCTTCGCTATC	GTGACCTCAAGCACATCCTG	426

HwTKL1	ATGGAAATTCCGTTGTCGTC	TTCTCCACCTGTTCGGCTAC	346
HwTOM40	CGGCACCAATAACATCCTCT	CTCCACCTTGTCCGTCAACT	451
HwTUB2	TAGCTCGGGTTGTTCAGCTT	CCGTATGATGGCCACTTTCT	183
HwUGP1	TGATGGTCAAGTGGTCAAGC	GAGGCGGACCTGAGCATC	301
SOL11	CTACAGCGAGGACATCACCA	CTGCAGCTTCACCTGTTCTG	319
SOL13	CTGCCAGATAACGACGAGGT	GACACCACCCACGACATCTA	252
SOL15	CTTGCTGACAGCCCAAGAG	ACTGCCAACGCGGCTTAC	228
SOL16	GGGCTCATGGAGAACAGAAA	GACGGTCTAATCCCAGCTCA	303
SOL17	GTTGTGGACGATGTTGTTGC	ACAGCTCCTTCGACATCACC	365
SOL18	CATTACTCTGACCCGCACCT	CCAGCAGTGTTGGTGAAAGA	390
SOL19	CCGTAGCACTCGGGTAGACA	CTGGTTTGACGACGGGTACT	264
SOL20	CCAGCAGTGTTGGTGAAAGA	GATCATCCTCTGCTCCGTTC	166
SOL21	TCATCAGCTCGTTGATTTCG	ATCTACGCAACCGCTACACC	204
SOL23	ACAGCGATTAGATCGGCAAA	GCCCGTTTCCGATAGAGC	238
SOL24	CAACACGCTGTCACCTGTCT	CATCCTCTTCAGTGGCTTCC	209
SOL25	CGACATGTTGTCGATATCTGCT	TTGATGACCATTAATCAACCACT	204
SOL26	GCCAAGGGCATTGAGTAAAA	GCCCTCCATATCTCGTTTCC	204
SOL27	CACTGTCTGCAACTACTACGAAGC	CGGCCGAGGTACATTGTTAT	350
SOL28	GACCGTGAGCTCAATCCTGT	GACCCTGTGCTTCAGAGACC	232
